# Recurrent mutations of BRCA1, BRCA2 and PALB2 in the population of breast and ovarian cancer patients in Southern Poland

**DOI:** 10.1186/s13053-016-0046-5

**Published:** 2016-02-03

**Authors:** P. Wojcik, M. Jasiowka, E. Strycharz, M. Sobol, D. Hodorowicz-Zaniewska, P. Skotnicki, T. Byrski, P. Blecharz, E. Marczyk, I. Cedrych, J. Jakubowicz, J. Lubiński, V. Sopik, S. Narod, P. Pierzchalski

**Affiliations:** Oncogene Diagnostics, Mogilska 86, 31-546 Kraków, Poland; Department in Cracow, Oncology Cancer Center Institute Marie Skłodowska-Curie, Cracow, Poland; First Chair of General, Oncological, and Gastrointestinal Surgery, Jagiellonian University Medical College, Krakow, Poland; Outpatient Clinic of Oncological Surgery, St. Raphael Hospital, Cracow, Poland; Faculty of Health Science, Department of Medical Physiology, Jagiellonian University Medical College, Cracow, Poland; Clinic of Oncology, Pomeranian Medical University, International Hereditary Cancer Center, Szczecin, Poland; Pomeranian Medical University, International Hereditary Cancer Center, Szczecin, Poland; Women’s College Research Institute, Women’s College Hospital, Toronto, Canada

**Keywords:** BRCA1, BRCA2, PALB2, Breast cancer, Ovarian cancer

## Abstract

**Background:**

Mutations in the BRCA1, BRCA2 and PALB2 genes are well-established risk factors for the development of breast and/or ovarian cancer. The frequency and spectrum of mutations in these genes has not yet been examined in the population of Southern Poland.

**Methods:**

We examined the entire coding sequences of the BRCA1 and BRCA2 genes and genotyped a recurrent mutation of the PALB2 gene (c.509_510delGA) in 121 women with familial and/or early-onset breast or ovarian cancer from Southern Poland.

**Results:**

A BRCA1 mutation was identified in 11 of 121 patients (9.1 %) and a BRCA2 mutation was identified in 10 of 121 patients (8.3 %). Two founder mutations of BRCA1 accounted for 91 % of all BRCA1 mutation carriers (c.5266dupC was identified in six patients and c.181 T > G was identified in four patients). Three of the seven different BRCA2 mutations were detected in two patients each (c.9371A > T, c.9403delC and c.1310_1313delAAGA). Three mutations have not been previously reported in the Polish population (BRCA1 c.3531delT, BRCA2 c.1310_1313delAAGA and BRCA2 c.9027delT). The recurrent PALB2 mutation c.509_510delGA was identified in two patients (1.7 %).

**Conclusions:**

The standard panel of BRCA1 founder mutations is sufficiently sensitive for the identification of BRCA1 mutation carriers in Southern Poland. The BRCA2 mutations c.9371A > T and c.9403delC as well as the PALB2 mutation c.509_510delGA should be included in the testing panel for this population.

## Background

Inherited mutations in the DNA repair genes BRCA1 and BRCA2, discovered and cloned almost 20 years ago, are associated with significantly increased risks of both breast and ovarian cancer [1–6]. The BRCA1/2 proteins participate in molecular repair pathways in response to DNA damage; BRCA1 is involved in the recognition of DNA damage and the regulation of cell cycle arrest and BRCA2 regulates the key homologous recombination enzyme Rad51 [7–14]. In addition to BRCA1/2, several other genes have been identified which predispose women to breast and/or ovarian cancer. Genes with high penetrance (i.e. relative risk of four or more) include TP53, PTEN and PALB2 [15–17]. Genes with moderate or low penetrance (i.e. relative risk of 1.5 to 3) include ATM, CHEK2, NBS1, and BRIP1 [18–21].

In Poland, a relatively small number of founder mutations in genes of high or moderate penetrance account for the majority of breast and/or ovarian cancer families. Among them are six mutations of BRCA1 (c.68_69delAG, c.181 T > G, c.3700_3704del, c.4035delA, c.5266dup and c.5251C > T) of BRCA1 mutations, a single mutation of NBS1 (c.657_661delACAAA) and two recently described mutations of PALB2 (c.509_510delGA and c.172_175delTTGT) [21–23]. Some recurrent mutations of BRCA2 have been reported (c.658_659delGT, c.3847_3848delGT, c.5239_5240insT, c.5946delT and c.7913_7917del), but the frequency of these mutations in Polish families is relatively low [19, 23, 24]. Kluska et al. recently proposed that the testing panel of Polish founder mutations be expanded to include two new mutations, c.1687C > T in BRCA1 and c.9371A > T in BRCA2 with the status pathogenic and likely pathogenic respectively, which showed strong founder effects in Polish patients with familial or early-onset breast and/or ovarian cancer [25]. Although the set of the most frequent deleterious mutations described above is generally accepted, there have been significant discrepancies in the frequency of recurrent mutations reported in different regions of Poland. Based on the data published previously by different Polish groups, we have decided that the set of five BRCA1 mutations (c.68_69delAG, c.181 T > G, c.3700_3704del, c.4035delA, c.5266dup), which is covering approximately 90 % of all detected in BRCA1 mutations associated with familial breast and ovarian cancer syndrome might be treated as a “standard panel” for Polish population [21, 23, 26–28].

The aim of our study was to identify the optimal panel of founder mutations for the genetic screening of patients at high risk for hereditary breast and/or ovarian cancer in Southern Poland. We compared the frequency and spectrum of BRCA1 and BRCA2 mutations contributing to breast and ovarian cancer families in Malopolska with those that have been previously reported in Polish families. We also examined the frequency of the recurrent PALB2 deletion c.509_510delGA in this population.

## Methods

### Patient group

We studied 121 women diagnosed with breast or ovarian cancer from families fulfilling one of the following criteria: 1) three or more breast and/or ovarian cancer cases in first or second-degree relatives, 2) synchronous or metachronous breast and/or ovarian cancer in the same patient, 3) two breast cancer cases in first-degree relatives (at least one diagnosed before age 50) or 4) breast cancer diagnosed before age 40. All patients in the study were recruited between 2012 and 2014 from three oncology centers in Małopolska: (i) Oncology Cancer Center Institute Marie Skłodowska-Curie, Department in Kraków, (ii) Outpatient Clinic of Early Diagnosis and Treatment of Breast Disease, Collegium Medicum Jagiellonian University, Kraków and (iii) Outpatient Clinic of Oncological Surgery, St. Raphael Hospital, Kraków. Written consent for genetic testing was obtained for all patients. This study was approved by the Ethics Committee (decision No. 71/KBL/OIL/2012).

### DNA isolation

Genomic DNA was isolated from 200 ul of peripheral blood on Extractme silica columns (EURx) according to the manufacturer’s protocol. The quality and concentration of extracted DNA was assessed using NanoDrop 2000 (ThermoScientific).

### HRM analysis

The BRCA1 coding sequence with flanking intronic sequences were analyzed for all patients using the High Resolution Melting (HRM) method. The HRM method is a commonly used for the detection of single nucleotide substitutions and small oligonucleotide insertion or deletion variants and has been shown to be suitable for the detection of BRCA1 mutations [29–31]. Primers were designed to amplify a fragment no longer than 300 bp to enable mutation detection. Coding sequences longer than 300 bp were covered with amplicons with overlapping flanking sequences. Optimal annealing temperature was established employing gradient PCR. Agarose gels were run for each primer set to ensure that the correct size product was produced and that there was no formation of non-specific products. The thermal cycling profile consisted of a three minute initial activation at 95 °C, 45 3-step cycles (5 s denaturation at 95 °C, annealing for 10 s, 10 s extension at 72 °C) and a final hold at 72 °C for 10 s. For the HRM analysis, fluorescence data acquisition was set from 77 °C to 89 °C, increasing the temperature by 0.1 °C each step. 20 ng of genomic DNA was added to the PCR mix containing primers pair and SensiFAST HRM kit (Bioline). 42 amplicons were used to analyze the BRCA1 sequence. A known homozygous wild type control and a no-template control were also included in the analyzed sample sets. The analysis was performed on Rotor-Gene Q 5plex HRM Platform (Qiagen). Samples with an aberrant melting profile were sequenced using Sanger sequencing.

### DNA sequencing

The BRCA2 coding sequence with flanking intronic sequence and the PALB2 mutation c.509_510delGA were analyzed for all patients using Sanger sequencing. Amplicons were sequenced using BigDye Terminator v3.1 Cycle Sequencing Kit and 3130xl Genetic Analyzer (Applied Biosystems). Sequencing data were examined using Sequencing Analysis software 5.2 and compared to reference sequences. Nucleotide reference sequences were as follows: BRCA1 (NM_007294.3, NG_005905.2), BRCA2 (NM_000059.3, NG_012772.3), PALB2 (NM_024675.3, NG_007406.1). Each detected mutation was confirmed by independent analysis starting from DNA extraction.

## Results

We screened 121 women with familial and/or early-onset breast or ovarian cancer for mutations in BRCA1, BRCA2 and PALB2. The mean age of cancer diagnosis for all patients was 48.5 years (range 27–85 years). There were 115 women with breast cancer (mean age of diagnosis: 48.6 years) and six women with ovarian cancer (mean age of diagnosis: 46.7 years).

A mutation in one of the three genes was detected in 23 of 121 patients (19.0 %). A BRCA1 mutation was identified in 11 patients (9.1 %) and a BRCA2 mutation was identified in ten patients (8.3 %). The PALB2 mutation c.509_510delGA was identified in two patients (1.7 %). Molecular and clinical characteristics of the mutation-positive patients are presented in Table 1.

**Table 1:** Molecular and clinical characteristics of mutation-positive patients

Patient	Type of cancer	Mutation	Age of onset	Number of ovarian cancers in family	Number of breast cancers in family
BRCA1					
1	Breast cancer	c.181 T > G, p.C61G “standard panel” mutation	43	2	0
2	Bilateral breast		35/42	0	6
3	Breast cancer		42	2	0
4	Breast cancer		25	2	2
5	Ovarian cancer	c.3531delT, p.F1177Lfs*33	39	1	0
6	Breast cancer	c.5266dupC, p.Q1756Pfs*74 “standard panel” mutation	59	3	0
7	Ovarian cancer		59	4	0
8	Breast cancer		44	0	1
9	Breast cancer		55	1	0
10	Breast cancer		44	1	1
11	Bilateral breast		25	1	0
BRCA2					
1	Breast cancer	c.1310_1313delAAGA, p.Lys437Ilefs*22	37	0	1
2	Breast cancer		38	0	4
3	Breast cancer	c.6267_6269delinsC, p.E2089_H2090delinsDfs*2	41	0	3
4	Bilateral breast	c.7913_ 7917delTTCCT, p.F2638*	40	0	1
5	Breast cancer	c.9027delT, p.Y3009Yfs*19	52	1	1
6	Breast cancer	c.9371 A > T, p.N3124I	67	1	1
7	Bilateral breast		-	2	1
8	Bilateral breast	c.9403delC, p.L3135Ffs*28	36	0	1
9	Breast cancer		37	2	1
10	Breast cancer	c.10095delCinsGAATTATATCT, p.V3365Vfs*5 (non-pathogenic)	61	0	1
PALB2					
1	Breast cancer	c.509_510delGA	<50	0	1
2	Breast cancer		48	0	2

Two common founder mutations of BRCA1 accounted for 91 % of all BRCA1 mutation carriers. The BRCA1 founder mutation c.5266dupC was identified in six patients (54.5 % of all BRCA1 carriers), and the BRCA1 founder mutation c.181 T > G was identified in four patients (36.4 % of all BRCA1 carriers). One patient was found to carry a rare BRCA1 mutation c.3531delT (9.1 % of all BRCA1 carriers). This variant has not been previously reported in the Polish population (Table 2).

**Table 2:** Frequency of rare (non-founder) BRCA1 mutations reported in the Polish population

BRCA1 mutation	Perkowska (2003) [39]	Górski (2004) [32]	Brożek (2008) [35]	Ratajska (2008) [26]	Rzepecka (2012) [54]	Gaj (2012) [55]	Cybulski (2014) [24]	Ratajska (2015) [27]	Szwiec (2015) [49]	Kluska (2015) [2015]	Current study
	N = 60	*N* = 200	*n* = 151	*N* = 64	*n* = 257	*n* = 906	*n* = 144	*n* = 134	*n* = 1164	*n* = 512	*n* =121
c.342_343delTC	-	-	-	-	-	-	-	-	-	1	-
c.405_408delAAGA	-	-	-	-	-	-	-	-	-	1	-
c.406delA	-	-	-	-	-	-	-	-	-	1	-
c.594-2A > C	-	-	-	-	-	-	-	1	-	-	-
c.676delT	-	2	-	-	-	-	-	-	-	-	-
c.1612C > T	-	-	-	-	-	-	-	-	-	1	-
c.1687C > T	-	-	2	-	-	-	1	-	-	8	-
c.1695_1696insG	-	-	-	-	-	-	-	-	-	1	-
c.1793 T > A	-	-	-	-	-	-	-	1	-	-	-
c.2563C > T	1	-	-	-	-	-	-	-	-	-	-
c.2866_2870delTCTCA	-	1	-	-	-	-	-	-	-	-	-
c.2872_2876delTTCAG	-	-	-	1	-	-	-	-	-	-	-
c.3531delT	-	-	-	-	-	-	-	-	-	-	1
c.3748G > T	-	-	-	-	1	-	-	-	-	-	-
c.3756_3759delGTCT	-	-	-	-	3	4	-	-	-	-	-
c.3779delT	-	-	-	-	-	2	-	-	-	-	-
c.3817C > T	-	-	-	1	-	-	-	-	-	-	-
c.4041_4042delAG	-	-	-	-	-	1	1	-	-	-	-
c.4357 + 2 T > G	-	-	-	-	-	-	-	1	-	-	-
c.4484 + 1G > A	1	-	1	-	-	-	-	-	-	-	-
c.4516delG	-	-	-	-	-	-	-	-	-	4	-
c.4597_4598delGA	-	-	-	-	-	-	-	-	-	1	-
c.4689C > G	-	-	-	-	-	-	-	-	-	1	-
c.5030_5033delCTAA	-	1	-	-	-	-	-	-	-	-	-
c.5095C > T	-	-	-	-	-	-	-	-	-	2	-
c.5137delG	-	-	-	-	-	-	-	-	-	1	-
c.5177_5180delGAAA	-	-	-	-	-	-	1	-	-	-	-
c.5232del7ins12	-	-	-	-	-	-	-	-	-	1	-
c.5251C > T	-	3	-	-	-	8	1	-	3	-	-
c.5289insG	-	-	-	-	-	-	-	-	-	1	-
c.5346G > A	1	-	-	-	-	-	1	-	-	2	-

n – represents number of patients included to the study; N – represents number of families included to the study

In the group of 10 patients harboring BRCA2 mutations, seven different single mutations have been detected. Three mutations (c.1310_1313delAAGA, c.9371A > T and c.9403delC) were identified in two patients each and four mutations (c.6267_6269delinsC, c.9027delT, c.7913_7917delTTCCT and c.10095delCinsGAATTATATCT) were identified in one patient each. Two of the BRCA2 mutations (c.1310_1313delAAGA and c.9027delT) have not been previously reported in the Polish population (Table 3).

**Table 3:** Overview of mutations found in the BRCA2 gene in the Polish population

	Looij (2000) [52]	Grzybowska (2000) [34]	Kwiatkowska (2001) [42]	Perkowska (2003) [39]	Górski (2004) [32]	Brożek (2008) [35]	Ratajska (2008) [26]	Balabas (2010) [36]	Gaj (2012) [55]	Cybulski (2014) [24]	Ratajska (2015) [27]	Szwiec (2015) [22]	Kluska (2015) [25]	Current study
	*N = 25*	*N = 47*	*n = 37*	*N = 60*	*N = 200*	*n = 151*	*N = 64*	*n = 105*	*n = 906*	*n = 144*	*n = 134*	*n = 1164*	*n = 512*	*n = 121*
c.2 T > C	-	-	-	-	-	-	-	-	-	1	-	-	-	-
c.22_23delAG	-	-	-	-	-	-	-	-	-	1	-	-	-	-
c.262_263delCT	-	-	-	-	1	-	-	-	-	-	-	-	-	-
c.274C > T	-	-	-	-	-	-	-	-	-	-	-	-	1	-
c.658_659delGT	-	-	-	-	-	-	-	-	-	-	-	1	-	-
c.700delT	-	-	-	-	-	-	-	-	-	-	-	-	1	-
c.1180G > T	-	-	-	-	-	-	-	1	1	-	-	-	-	-
c.1310_1313delAAGA	-	-	-	-	-	-	-	-	-	-	-	-	-	2
c.1318_1321delCTTA	-	-	-	-	-	-	-	-	-	-	-	-	1	-
c.1813_1814insA	-	-	1	-	-	-	-	-	-	-	-	-	-	-
c.2806_2809delAAAC	-	-	-	-	-	-	-	-	-	1	-	-	-	-
c.2808_2811delACAA	-	-	-	-	-	-	-	-	-	-	1	-	1	-
c.3199delA	-	-	-	-	1	-	-	-	-	-	-	-	-	-
c.3847_3848delGT	1	-	-	-	-	-	-	-	-	-	-	1	-	-
c.3860delA	-	-	-	1	-	-	-	-	-	-	-	-	-	-
c.3975_3981dupTGCT	-	-	-	-	-	1	-	-	-	1	1	-	-	-
c.4223delA	-	-	-	-	-	1	-	-	-	-	-	-	-	-
c.5042_5043delTG	-	-	-	-	-	-	-	-	-	-	1	-	-	-
c.5239_5240insT	-	-	-	-	1	-	-	1	4	-	-	1	-	-
c.5722_5723delCT	-	-	-	-	-	-	-	-	-	1	-	-	-	-
c.5946delT	-	-	-	-	-	-	-	1	4	-	-	1	-	-
c.5964_5965delAT	-	-	-	-	-	-	-	1	-	-	-	-	-	-
c.6010_6030delinsTT	-	-	-	-	-	-	1	-	-	-	-	-	-	-
c.6267_6269delGCAinsC	-	-	1	-	-	-	-	-	-	-	-	-	-	1
c.6275_6276delTT	-	-	-	-	-	-	-	-	-	1	-	-	-	-
c.6393_6396delATTA	-	-	-	-	-	1	-	-	-	-	-	-	-	-
c.6402_6406delTAACT	-	-	-	-	-	-	-	-	-	1	-	-	-	-
c.6447_6448delTA	-	-	-	-	-	-	-	1						
c.6658_6662delGAAAA	-	1	-	-	-	-	-	-	-	-	-	-	-	-
c.7007G > A	-	-	-	-	1	-	-	-	-	-	-	-	-	-
c.7180A > T	-	-	-	-	-	-	-	-	-	-	1	-	-	-
c.7251_7252delCA	-	-	-	-	-	-	-	-	-	1	-	-	1	-
c.7080_7099dup	1	-	-	-	-	-	-	-	-	-	-	-	-	-
c.7913_7917delTTCCT	-	-	1	-	1	-	-	3	2	-	-	1	-	1
c.8648delC	-	-	-	-	-	-	1	-	-	1	-	-	-	-
c.8842delA	1	-	-	-	1	-	-	-	-	-	-	-	1	-
c.8924delT	-	-	-	-	-	-	-	1	1	-	-	-	-	-
c.8946delA	-	-	-	-	-	-	-	-	-	-	-	-	1	-
c.9027delT	-	-	-	-	-	-	-	-	-	-	-	-	-	1
c.9097_9098insA	-	-	-	-	-	-	-	1	-	1	-	-	-	-
c.9118-2A > G	-	-	-	-	-	-	-	1	-	-	-	-	3	-
c.9138C > T	-	-	-	-	-	-	-	-	-	-	-	-	1	-
c.9253_9254insA	-	-	-	-	-	-	-	-	-	1	-	-	-	-
c.9371A > T	-	1	1	-	-	-	-	3	-	-	-	-	6	2
c.9376delC	-	-	-	-	-	-	-	-	-	-	-	-	1	-
c.9403delC	-	3	-	-	1	-	-	1	-	1	-	-	-	2
c.10095delCinsGAATTATATCT	-	-	-	-	-	-	2	1	-	-	-	-	-	1

n – represents number of patients included to the study; N – represents number of families included to the study

In addition to the pathogenic mutations described above, several synonymous and non-synonymous variants were detected in the analyzed genes, including ten in BRCA1 (c.734A > T, c.1067A > G, c.2077 G > A, c.2082C > T, c.2311 T > C, c.3113A > G, c.3548A > G, c.4039A > G, c.4308 T > C, c.4837A > G), nine in BRCA2 (c.3396 A > G, c.3516 G > A, c.3807 T > C, c.5199 C > T, c.5744C > T, c.7242A > G, c.8182G > A, c.8850G > T, c.10202C > T) and one in PALB2 (c.1010 T > C).

Two of the 11 BRCA1 mutations were identified in women with high-grade serous ovarian cancer and nine were identified in women with breast cancer. Of the six BRCA1 mutation-associated breast cancers with information on histopathology, hormone receptor status and HER2 status, four were triple-negative (ER/PR/HER2-negative) invasive ductal carcinomas and two were atypical medullary breast cancers (ER/PR-positive, HER2-negative). All nine BRCA2 mutations were identified in women with breast cancer. Of the eight BRCA2 mutation-associated breast cancers with information on histopathology, hormone receptor status and HER2 status, five were ER/PR-positive, HER2-negative invasive ductal carcinomas and three were ER/PR-positive, HER2-positive invasive ductal carcinomas. Both PALB2 mutation-associated breast cancers were ER/PR-positive invasive ductal carcinomas with coexisting ductal carcinoma in situ.

## Discussion

We identified a BRCA1/2 mutation in 21 (17.4 %) of 121 patients with familial and/or early-onset breast or ovarian cancer from Southern Poland. A BRCA1 mutation was identified in 11 patients (9.1 %) and a BRCA2 mutation was identified in ten patients (8.3 %). The prevalence of BRCA1/2 mutations is relatively low compared to previous studies of Polish breast and/or ovarian cancer families, in large part due to the low frequency of BRCA1 mutations. In a study of 200 Polish families with three or more breast or ovarian cancers a BRCA1/2 mutation was identified in 64 %; the prevalence of BRCA1 mutations was 60 % and the prevalence of BRCA2 mutations was 4 % [32]. In a study of Northern Polish families with similar characteristics as the families in our study, a BRCA1/2 mutation was identified in 67 %; the prevalence of BRCA1 mutations was 61 % and the prevalence of BRCA2 mutations was 6 % [26]. The relatively low frequency of BRCA1 mutations reported here may be explained by a lower prevalence of mutations in this particular region of Poland, noticed and reported previously by Blecharz et al and Brozek et al [28, 33]. The frequency of BRCA2 mutations in this study (8.3 %) is within the range of mutation frequencies reported in previous studies of Polish families (2.6 to 11.4 %) [24, 34–37].

The two most frequent mutations identified in the present study were the BRCA1 founder mutations c.5266dupC (identified in six patients) and c.181 T > G (identified in four patients). These two founder mutations accounted for 91 % of all BRCA1 mutation carriers, which in general is consistent with previous studies in the Polish population (Table 4) [26, 33, 38, 39]. However, in our study BRCA1 founder mutations accounted for only 48 % of all BRCA1 and BRCA2 mutation carriers, whereas in previous studies BRCA1 founder mutations accounted for 80 to 90 % of all BRCA1/2 carriers [26, 28, 32]. In most previous studies there was a preponderance of BRCA1 mutations over BRCA2 mutations. In our study, the frequency of BRCA1 and BRCA2 mutations is similar, and BRCA2 mutations make a larger contribution to all BRCA1/2 mutation carriers. The relatively low ratio of BRCA1 to BRCA2 mutations reported here may be due to the low prevalence of BRCA1 founder mutations in this region (discussed above) or perhaps there has not been enough BRCA2 mutation screening in Poland because of the lack of founder mutations and more research is warranted. Nevertheless, it is of particular importance to identify recurrent mutations of BRCA2 in this population.

**Table 4:** Basic panel (“standard panel”) of the five most common mutations identified in BRCA1 and their relative frequency in the Polish population

Mutation	Looij (2000) [52]	Grzybowska (2000) [34]	Grzybowska (2002) [53]	Perkowska (2003) [39]	Górski (2004) [32]	Górski (2006) [38]	Brożek (2008) [35]	Ratajska (2008) [26]	Blecharz (2009) [28]	Rzepecka (2012) [54]	Gaj (2012) [55]	Szwiec (2015) [22]	Ratajska (2015) [27]	Current study
c.5266dupC	20 %	92 %	73 %	55 %	59 %	55 %	46 %	57 %	57 %	62 %	66 %	52 %	62 %	60 %
c.181 T > G	60 %	8 %	13 %	27 %	27 %	21 %	8 %	24 %	29 %	16 %	22 %	25 %	15 %	40 %
c.4035delA	0 %	0 %	0 %	0 %	10 %	12 %	8 %	0 %	8 %	5 %	1 %	4 %	0 %	0 %
c.3700_3704delGTAAA	20 %	0 %	0 %	9 %	3 %	6 %	31 %	11 %	6 %	14 %	6 %	12 %	23 %	0 %
c.68_69delAG	0 %	0 %	13 %	9 %	1 %	6 %	8 %	8 %		3 %	5 %	7 %	0 %	0 %

Three of the seven different BRCA2 mutations identified in the current study were recurrent (i.e. found in more than one patient). Two patients carried the likely pathogenic BRCA2 mutation c.9371A > T. This substitution is located in an evolutionarily conserved region of the BRCA2 gene and is predicted to adversely affect ssDNA interaction and the structural integrity of the C-terminal domain of the protein [40, 41]. This variant has previously been reported in 11 unrelated patients from Poland (Table 3) [25, 34, 36, 42]. Kluska et al recently identified the variant in six patients and proposed its inclusion in the list of Polish founder mutations [25]. Two patients carried the BRCA2 mutation c.9403delC. This mutation has been previously reported in six unrelated patients from Poland (Table 3), including three patients from Southern Poland [34]. The mutation has also been reported in patients from the Czech Republic and Slovakia [43, 44]. This variant may be a local founder mutation and should be included as a part of the screening test for the Polish population. Two patients carried the BRCA2 mutation c.1310_1313delAAGA. This mutation has not been previously reported in other studies from Poland. However, it has been frequently reported in Denmark and less frequently in France, Spain and North Africa [45–47]. The prevalence of this mutation in Southern Poland should be examined further. Including the two recurrent BRCA2 mutations that have been previously identified in the Polish population (c.9371A > T and c.9403delC) in the standard testing panel would increase the sensitivity of the panel for all BRCA1/2 mutation carriers from 48 to 67 %.

One patient carried the BRCA2 frame-shift variant c.10095delCinsGAATTATATCT. This variant truncates the BRCA2 protein at the very end of the C-terminus and according to the BIC database is most likely benign. We classified this variant as non-pathogenic, however there is one previous report which suggests a potential association of the variant with familial aggregation of breast and/or ovarian cancer [48]. Furthermore, in our family, the variant was identified in two first-degree relatives with breast cancer, including one patient who was diagnosed before age 50. This variant has now been identified in four unrelated Polish patients (Table 3).

We identified the PALB2 mutation c.509_510delGA in two unrelated patients (1.7 %), both of whom were diagnosed with breast cancer before age 50 and had one affected relative. This mutation was recently identified in 70 (0.6 %) of 12,529 unselected breast cancer patients from Poland, and is considered to be a recurrent mutation in the Polish population [49, 50]. In a study of 144 Polish breast cancer families, the mutation was identified in 1.4 % and accounted for 50 % of all PALB2 mutation carriers [23]. The full spectrum of PALB2 mutations in the population of southern Poland is not known. The prevalence of this mutation in unselected breast cancer patients in the neighboring countries is as follows: Germans 0.3 %, Russians 0.2 % and Belarusians 0.3 % [51].

PALB2 c.509_510delGA is one of two recurrent PALB2 mutations that have been recently described in the Polish population. The other mutation, c.172_175delTTGT, has been identified in 0.3 % of unselected breast cancer patients and 0.7 % of familial breast cancer patients from Poland [23, 49]. Among families with three or more breast cancers, the detection rate of 509_510delGA and 172_175delTTGT PALB2 mutations increases to 4.6 %. The risk of developing breast cancer is more than four times higher in PALB2 mutation carriers compared to non-carriers. Among those who develop breast cancer, the risk of dying at ten years is two times higher for PALB2 mutation carriers compared to non-carriers (HR = 2.3; *p* < 0.0001).

In summary, we have identified five recurrent mutations in women with familial and/or early-onset breast and/or ovarian cancer from Southern Poland; BRCA1 c.5266dupC in six patients (prevalence: 5 %), BRCA1 c.181 T > G in four patients (prevalence: 3.3 %), BRCA2 likely pathogenic c.9371A > T in two patients, (1.7 %), BRCA2 c.9403delC in two patients (1.7 %) and PALB2 c.509_510delGA in two patients (1.7 %). Including the three additional recurrent mutations to the standard testing panel of BRCA1 founder mutations would increase the mutation detection rate from 8.3 to 13.4 %.

## Conclusions

Based on the results presented above and data from previous studies we conclude that (i) the “standard panel” of five BRCA1 founder mutations, described published previously by different Polish groups is sufficiently sensitive to identify BRCA1 mutation carriers in a screening test and (ii) the recurrent BRCA2 mutations N3124I and 9631delC as well as the recurrent PALB2 mutation c.509_510delGA could be included in the testing panel for the Southern Polish population. The proposed enlargement of the mutation set used for routine screening for familial and/or early-onset breast and/or ovarian cancer might turn-out to be beneficial for the patients.
